# Chemical and structural characterization of α-N-acetylgalactosaminidase I and II from starfish, *asterina amurensis*

**DOI:** 10.1186/s12858-017-0085-1

**Published:** 2017-05-25

**Authors:** Md. Harun-Or Rashid, Golam Sadik, AHM Khurshid Alam, Toshihisa Tanaka

**Affiliations:** 1 Institute of Biological Science, University of Rajshahi, Rajshahi, 6205 Bangladesh; 2 Department of Pharmacy, University of Rajshahi, Rajshahi, 6205 Bangladesh; 3 Department of Psychiatry, Osaka University Graduate School of Medicine, Osaka, 565-0871 Japan

**Keywords:** Characterization, α-N-Acetylgalactosaminidase, α-galactosidase, Starfish

## Abstract

**Background:**

The marine invertebrate starfish was found to contain a novel α-N-acetylgalactosaminidase, α-GalNAcase II, which catalyzes removal of terminal α-N-acetylgalactosamine (α-GalNAc), in addition to a typical α-N-acetylgalactosaminidase, α-GalNAcase I, which catalyzes removal of terminal α-N-acetylgalactosamine (α-GalNAc) and, to a lesser extent, galactose. The interrelationship between α-GalNAcase I and α-GalNAcase II and the molecular basis of their differences in substrate specificity remain unknown.

**Results:**

Chemical and structural comparisons between α-GalNAcase I and II using immunostaining, N-terminal amino acid sequencing and peptide analysis showed high homology to each other and also to other glycoside hydrolase family (GHF) 27 members. The amino acid sequence of peptides showed conserved residues at the active site as seen in typical α-GalNAcase. Some substitutions of conserved amino acid residues were found in α-GalNAcase II that were located near catalytic site. Among them G171 and A173, in place of C171 and W173, respectively in α-GalNAcase were identified to be responsible for lacking intrinsic α-galactosidase activity of α-GalNAcase II. Chemical modifications supported the presence of serine, aspartate and tryptophan as active site residues. Two tryptophan residues (W16 and W173) were involved in α-galactosidase activity, and one (W16) of them was involved in α-GalNAcase activity.

**Conclusions:**

The results suggested that α-GalNAcase I and II are closely related with respect to primary and higher order structure and that their structural differences are responsible for difference in substrate specificities.

## Background

Alpha-N-acetylgalactosaminidase (α-GalNAcase) [EC 3.2.1.49] is a lysosomal hydrolase that catalyzes the removal of terminal α-N-acteylgalactosamine (α-GalNAc) and, to a lesser extent, galactose monosaccharide from polysaccharides, glycolipids and glycoproteins [1]. The physiological importance of this enzyme is evidenced by the catabolic disorders due to deficiency of α-GalNAcase. In human, mutation in α-GalNAcase gene leads to a loss of enzymatic activity in the lysosome and subsequent accumulation of undegraded glycoconjugates in tissues [2], which eventually results in Schindler diseases and Kanzaki disease [3–6]. α-GalNAcase enzyme is most closely related to the α-galactosidase A (α-galactosidase) enzyme, and mutation in α-galactosidase A gene lead to Fabry disease [3, 4].

Because of similar substrate specificity and physical properties including subunit molecular mass and homodimeric structure, α-GalNAcase was initially referred to as α-galactosidase B. However, subsequent studies of kinetics, immunoreactivity, peptide and gene mapping demonstrated that α-GalNAcase and α-galactosidase are two distinct enzymes derived from two different genes [7]. Upon crystallographic analysis, they were found to have high similarity in active sites as 11 of the 13 active site residues are conserved, differing only in their recognition sequences. Base upon similarities in sequences, active sites, and mechanisms, both human α-GalNAcase and α-galactosidase are classified into family 27 of glycoside hydrolase family (GHF 27) [8].

α-GalNAcase has been purified from various animal tissues including human, bovine, pig, chicken, earthworm, snail and gastropod [7, 9–12]. Thus far, all the α-GalNAcases from animal tissues exhibited α-galactosidase activity. α-GalNAcase without α-galactosidase activity has been found only in microbes. Much efforts were directed for isolation of microbial α-GalNAcases that can efficiently cleave the immunodominant α-GalNAc residue from blood group A antigen at physiological pH. Prokaryotic α-GalNAcases are grouped into family GH 36 and GH 109 [13–17].

Marine animals are well known as potential sources of novel enzymes and provide useful information in understanding the structure and function of mammalian enzymes. We previously reported the occurrence of an unusual form, α-GalNAcase II, which was free from α-galactosidase activity, in addition to a typical form, α-GalNAcase I in squid (*Todarodes pacificus*), a marine invertebrate [18]. To confirm the presence of novel form is common in marine invertebrate, we further explored α-GalNAcases in starfish (*Asterina amurensis*) and purified two different α-GalNAcases, α-GalNAcase I and II. The two enzymes appeared to be different with respect to enzymatic characteristics such as molecular mass, optimum pH, pI, pH stability, enzyme kinetics (*K*_M_ and *V*max), and substrate specificities. α-GalNAcase II was monomer and heat stable, while α-GalNAcase I was multimer and heat labile. Both α-GalNAcase I and II removed terminal α-GalNAc of natural compounds investigated: Forsman hapten glycolipid, blood group A type trisaccharide, GalNAc-α1-O-serine. Contrary, oligosaccharide and glycolipid containing α-galactosyl terminal were hydrolyzed by α-GalNAcase I only, but not by α-GalNAcase II [19]. The *K*_M_ and *V*max values of α-GalNAcase II for pNP-α-GalNAc (3.97 mM and 126 μmole/min/mg) were much higher than that of α-GalNAcase I (1.04 mM and 49.5 μmole/min/mg). α-GalNAcase II activity was significantly inhibited by Ag^+^ and Hg^+2^ salts, while α-GalNAcase I activity was not much affected. However, the interrelationship between α-GalNAcase I and α-GalNAcase II and the molecular basis of their enzymological differences remain to be known.

In this study, we made chemical and structural comparisons between α-GalNAcase I and II from starfish using deglycosylation, immunostaining, N-terminal sequencing, peptide analysis and chemical modifications. In addition, we proposed the relationship between the substrate specificities and protein structures of the two enzymes.

## Results

### Homogeneity and deglycosylation of α-GalNAcase I and II

**Fig. 1:**
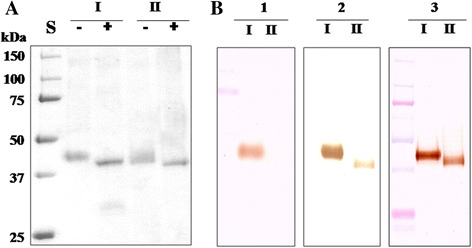
Deglycosylation and immunostaining of α-GalNAcase I and II. **a** SDS-PAGE of purified and deglycosylated α-GalNAcase I and II. The proteins were separated on SDS-PAGE and the gel was stained with CBB R-250 solution. S, Molecular weight marker; −, purified enzyme; +, enzyme treated with PNGase F. **b** Immunostaining between α-GalNAcase and anti-α-GalNAcase antibodies. 1, Antiserum to human α-GalNAcase; 2, antiserum to squid α-GalNAcase I; and 3, antiserum to squid α-GalNAcase II. Lane I, Enzyme α-GalNAcase I; and Lane II, Enzyme α-GalNAcase II

Two enzymes, α-GalNAcase I and II were purified from digestive organ of starfish and their enzymatic properties were characterized [19]. Both α-GalNAcase I and II appeared to be homogeneous and gave single protein band on SDS-PAGE at 47 kDa and 43 kDa subunit mass, respectively. The respective subunit mass of α-GalNAcase I and II was reduced by about 4 kDa upon treatment with PNGase F, indicating that both enzymes released the same extent of N-linked glycan (Fig. 1a). The deglycosylated protein showed sharp and narrow, while the glycosylated protein showed broad band.

### Immunological property

**Fig. 2:**
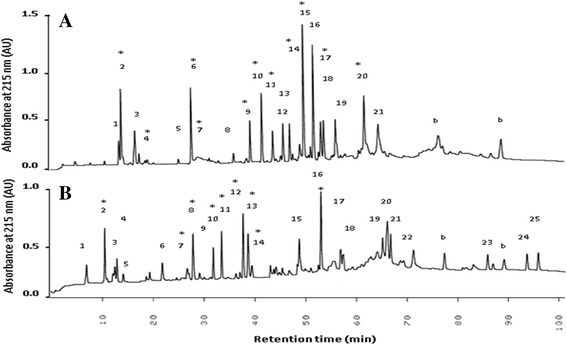
Peptide maps of α-GalNAcase I and II. RP-HPLC separation of lysylendopeptidase digested peptides of α-GalNAcase I **a** and α-GalNAcase-II **b**. Blank sample (without enzyme) contain the peaks b. The peptide peaks indicated with asterisk are subjected for amino acid sequence by MS/MS analysis. See text for details

**Fig. 3:**
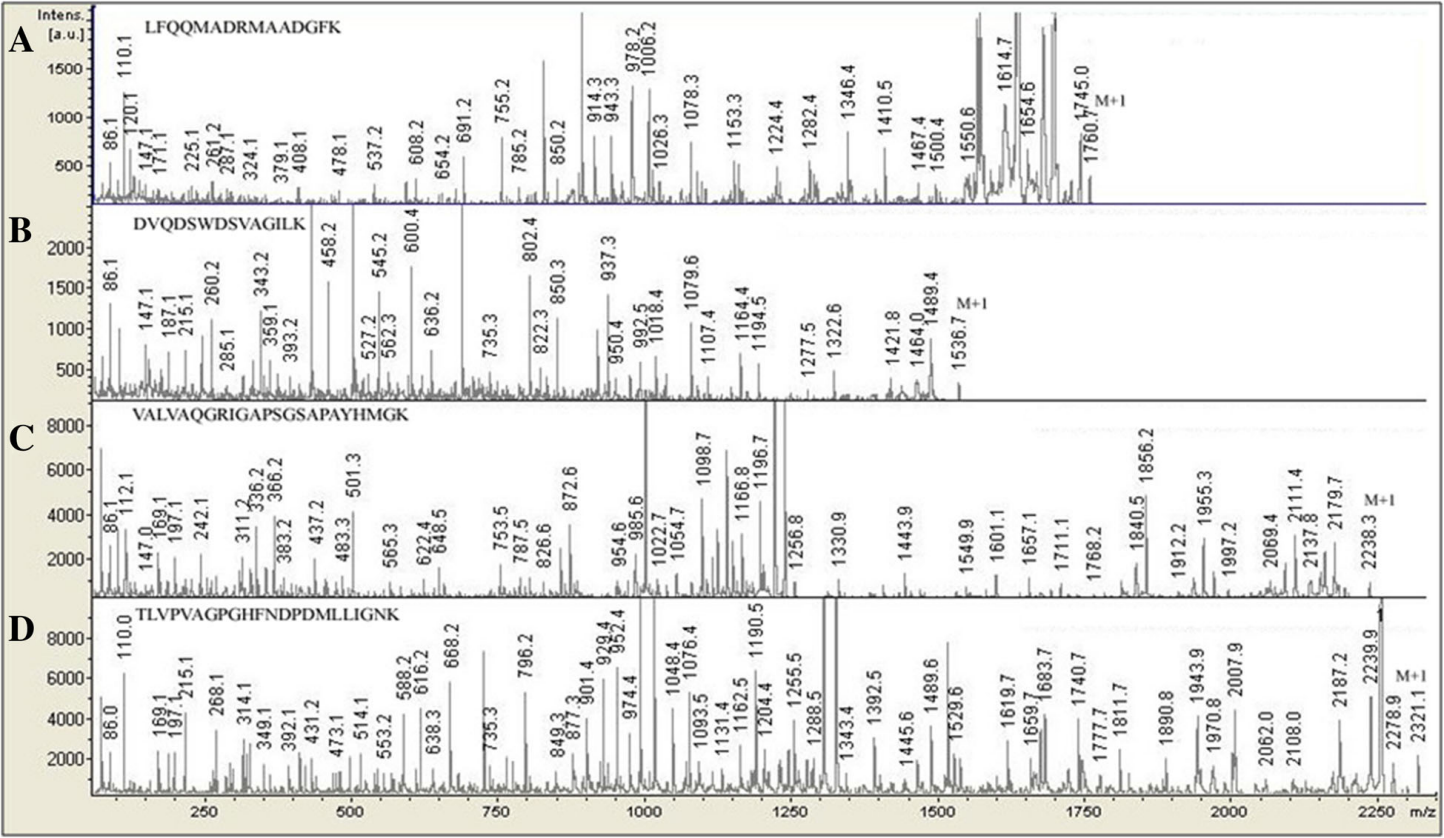
MS/MS analysis for amino acid sequence of the peptides of α-GalNAcase I and II. The individually obtained peptide fraction from RP-HPLC were subjected to MS/MS fragmentation. Amino acid sequences of peptides were determined from the fragmentation pattern by Bruker Data Analysis software (version 4.0). **a**, Peptide 17 of enzyme I; **b**, peptide 15 of enzyme I; **c**, peptide 13 of enzyme II; **d** peptide 16 of enzyme II

To study structural homologies of α-GalNAcase I and II, immunological techniques were applied using anti-human α-GalNAcase polyclonal anti-body produced in rabbit [20]. Interestingly, anti-human α-GalNAcase reacted intensely with α-GalNAcase I and showed no detectable cross reaction with α-GalNAcase II (Fig. 1b-1), suggesting that α-GalNAcase I and II might have different antigenicity. Moreover, we used anti-squid α-GalNAcase produced in rabbit [20] in immunostaining to differentiate immunological properties of α-GalNAcase I and II. Anti-α-GalNAcase I reacted intensely with α-GalNAcase I and barely with α-GalNAcase II (Fig. 1b-2), whereas, anti-α-GalNAcase II reacted equally with both α-GalNAcase I and II (Fig. 1b-3).

### Structural homology

The comparison between α-GalNAcase I and II were carried out by N-terminal sequencing of the first twenty amino acids. The deduced N-terminal sequences of α-GalNAcase I and II were compared with the corresponding sequences from human [21], chicken [22] and squid α-GalNAcases [23], and human α-galactosidase [24]. Sequence similarity analysis revealed 75% homology between GalNAcase I and α-GalNAcase II. Both enzymes were highly homologous (80–90%) to human α-galactosidase, human and chicken α-GalNAcases and to more closely related α-GalNAcases from squid (Table 1).

**Table 1:** N-Terminal amino acid sequences of α-GalNAcase from starfish and other sources

Enzyme	Amino acid sequences																				Homology
	1	2	3	4	5	6	7	8	9	10	11	12	13	14	15	16	17	18	19	20	
Chicken liver	L	E	N	G	L	A	R	T	P	P	M	G	W	L	A	W	E	R	F	R	100%
Starfish I	L	D	N	G	L	A	L	T	P	P	M	G	W	L	S	W	E	R	T	R	80%
Starfish II	L	D	N	G	L	G	R	T	P	P	M	G	W	M	T	W	E	R	F	R	80%
Squid I	L	N	N	G	L	A	L	T	P	P	M	G	W	L	S	W	E	R	F	R	85%
Squid II	L	D	N	G	L	M	R	T	P	P	M	G	W	L	V	W	E	R	F	M	80%
Human placenta	L	D	N	G	L	L	Q	T	P	P	M	G	W	L	A	W	E	R	F	R	85%
Hu-α-Gal	L	D	N	G	L	A	R	T	P	T	M	G	W	L	H	W	E	R	F	M	85%

Human placenta (Tsuji et al. [21]); Chicken liver (Zhu and Goldstein [22]), and Hu-α-Gal (Human α-galactosidase, Tsuji et al. [24]). Squid (Sadik et al. [23]). Numbering of amino acid started from N-terminal leucine of chicken α-GalNAcase

**Fig. 4:**
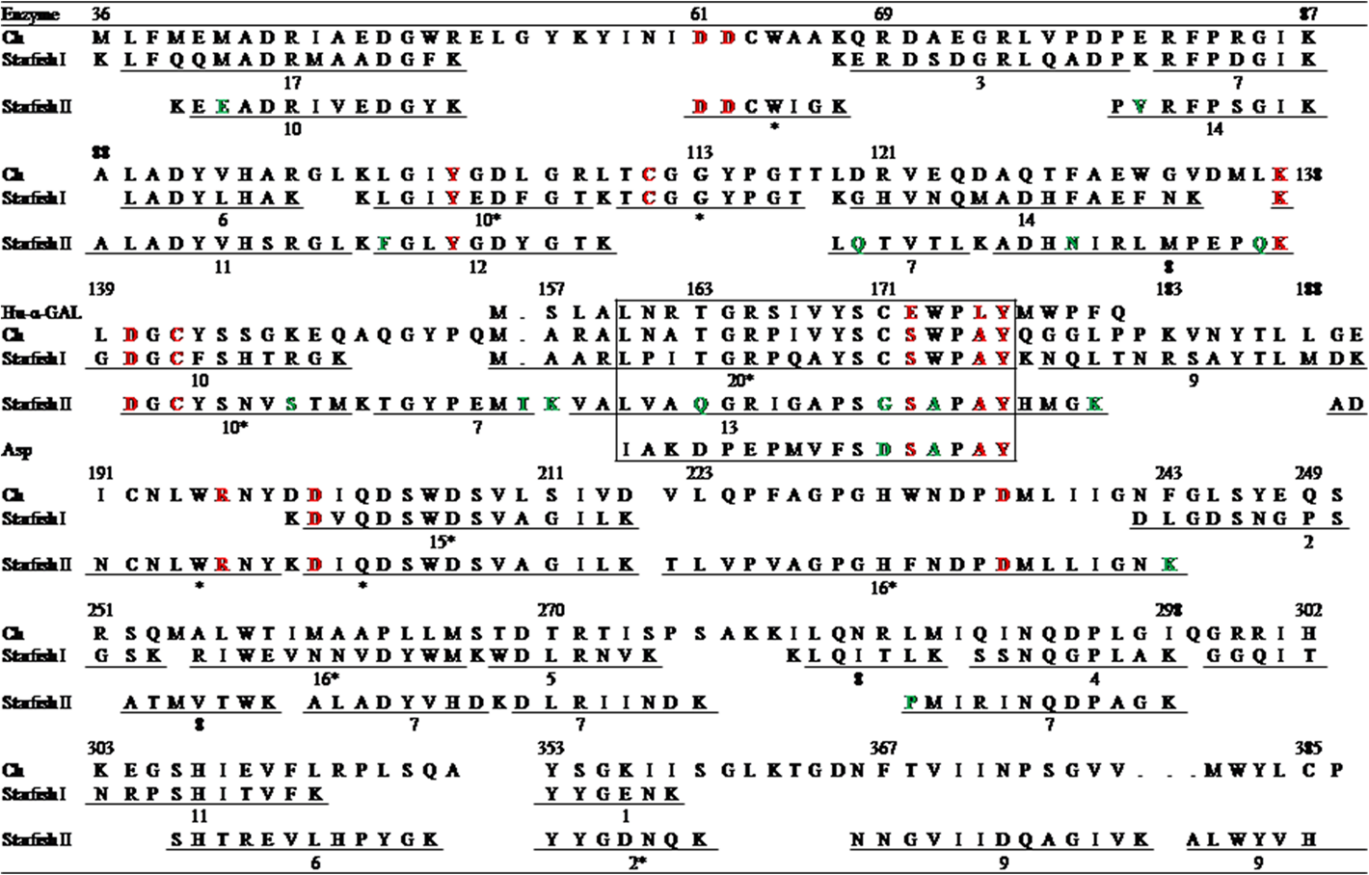
Summary of peptide sequences of α-GalNAcase I and II from starfish and alignment with that of chicken α-GalNAcases. The peptide sequences α-GalNAcase I and II were aligned with the corresponding sequence from chicken (Ch) α-GalNAcase. The underlined number indicated the respective peptide on Fig. 2. The peptide sequences marked with asterisk were analyzed by sequence analyzer. Numbering of amino acid started from N-terminal leucine of chicken α-GalNAcase. (Blank), means not determined the sequence. (.), means absent of amino acid residue. The conserved active site residues are shown in red. Boxed peptides comprise the N-acetyl recognition loop. The substitution of conserved residues in α-GalNAcase II as compared to typical α-GalNAcase are shown in green. Asp, *Acremonium species* α-GalNAcase (Ashida et al. [15]); Hu-α-Gal, Human α-galactosidase (Tsuji et al. [24])

The primary structures of α-GalNAcase I and II were also compared by peptide mapping. α-GalNAcase I and II were digested with lysylendopeptidase to have near completion for 24 h and resulting digestive products were separated by reversed-phase high performance liquid chromatography (RP-HPLC) (Fig. 2). Comparison of peptide maps of α-GalNAcase I and II revealed both similarities and differences in their peptide peaks. Most of the individually collected peptides were subjected to MS/MS analysis for sequencing and some of them are shown in Fig. 3. We could not confirm the amino acid sequence of each of the peptides due to either low yield or contamination with the other peptides. The peptide sequences of α-GalNAcase I and II, when aligned with the counterpart from chicken α-GalNAcase amino acid sequences, showed high homology as determined by BLAST homology search (Fig. 4). The active site residues (W16, D61, D62, Y103, C111, K138, D140, C142, S172, A175, Y176, R197 and D201) were conserved in both α-GalNAcase I and II. However, some substitutions of amino acid residues G6, M14, T15, E41, K78, F100, Q163, G171, A173, F232 were identified in α-GalNAcase II in place of the corresponding residues A6, L14, A15, M41, D78, L100, T163, C171, W173, and W232 in chicken α-GalNAcase (Table 1 and Fig. 4). Interestingly, two amino acid substitutions, G171 and A173 were found in α-GalNAcase II near the catalytic site that were conserved in α-GalNAcase I and other typical α-GalNAcases, suggesting that these residues might play important roles in determining the substrate specificity. From molecular modeling of the enzymes (details in methods), it appears that two disulphide bridge C111-C142 and C171-C193 exist in GalNAcase I near the catalytic site, whereas, one disulphide bridge (C171-C193) is absent in α-GalNAcase II due to substitution of C171 with G171.

### Inhibition of enzyme activity

**Fig. 5:**
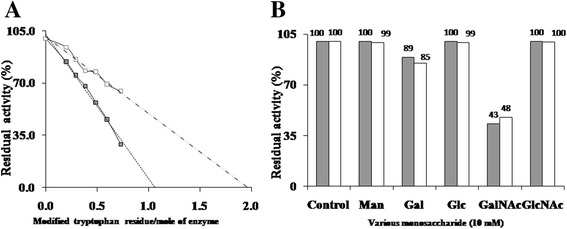
Effects of tryptophan modification and various monosaccharides on α-GalNAcase and α-galactosidase activities of α-GalNAcase I. **a** The NBS titration curve of percent residual activity versus the number of tryptophan residues modified was poltted for determination of number of tryptophan residues involved in α-GalNAcase and α-galactosidase activities of α-GalNAcase I. **b** Effect of various monosaccharide on α-GalNAcase I. α-GalNAcase I was preincubated with various monosaccharide (Man, Gal, Glc, GalNAc and GlcNAc) at 10 mM concentration for 30 min at 25 °C and determined the residual α-galactosidase (□) and α-GalNAcase (■) activities. Data are presented as mean value of three determinations

Chemical modification studies were carried out to confirm the active site residues involved in the catalytic reaction of α-GalNAcase. The involvement of serine, aspartate and tryptophan in the catalytic activity was evident by modification of these residues, which resulted in significant loss of enzyme activity of α-GalNAcase I and II, by DFP, CNM-HCl and NBS, respectively (Table 2). Modification of carboxyl group of glutamate by CNM-HCl is not responsible for inactivation of enzyme, because glutamate residue is not present in the catalytic site of α-GalNAcase [25, 26]. NBS specifically modified tryptophan rather tyrosine at low pH [27]. Like α-GalNAcase activity, the α-galactosidase activity of enzyme I was similarly inactivated by DFP and CNM-HCl. However, the retention of α-galactosidase activity was higher than that of α-GalNAcase activity in NBS mediated inactivation. To find out the reason for high retention of α-galactosidase activity of α-GalNAcase I, we determined the number of tryptophan residues involved in activity. NBS mediated inactivation of enzyme was accompanied by decrease in absorbance of modified protein at 280 nm. Using the molar absorption coefficient of tryptophan at 280 nm of 5500 M^−1^ cm^−1^, a graph of percent residual activity versus the number of tryptophan residues modified was plotted for α-GalNAcase and α-galactosidase activities of enzyme I (Fig. 5a). Extrapolation of graph to 0% activity confirms that α-GalNAcase activity depends on a single tryptophan residue and α-galactosidase activity depends on two tryptophan residues. These results indicate that tryptophan residue has a critical role in enzyme activity. In the inhibition studies with various monosaccharides, α-GalNAcase activity was found to be strongly inhibited by GalNAc and slightly by galactose, and similarly, the α-galactosidase activity of α-GalNAcase I was inhibited by those inhibitors as well (Fig. 5b).

**Table 2:** Effect of various selective amino acid modifying agents on α-GalNAcase and α-galactosidase activities of starfish α-GalNAcase I and II

Modifying agent		Residual activity (%)			Modified amino acid
Name	Conc. (mM)	Ia	Ib	II	
DFP	0	100.0	100.0	100.0	-
	3	85.7 ± 1.10	86.9 ± 1.53	77.1 ± 1.96	
	6	38.5 ± 0.84	41.1 ± 0.96	33.8 ± 1.21	Serine
	8	4.2 ± 0.35	4.9 ± 0.78	18.5 ± 0.45	
	10	0 ± 0.12	0 ± 0.49	3.5 ± 0.43	
CNM-HCl	10	97.4 ± 0.99	93.4 ± 1.56	95.6 ± 1.76	Aspartate
	30	37.9 ± 0.58	31.4 ± 2.43	36.0 ± 0.57	
	50	1.7 ± 0.32	1.7 ± 0.69	5.5 ± 0.48	
	60	0.4 ± 0.51	0.8 ± 0.65	2.2 ± 0.39	
NBS	0.005	48.5 ± 0.79	75.9 ± 1.78	54.4 ± 2.01	Tryptophan
	0.01	26.5 ± 0.65	52.4 ± 1.45	14.9 ± 0.97	
	0.02	10.0 ± 0.62	24.8 ± 1.09	7.6 ± 0.72	
	0.04	1.0 ± 0.87	2.8 ± 0.76	1.1 ± 0.23	

The residual activity was determined against pNP-α-GalNAc (for α-GalNAcase activity) and pNP-α-galactoside (for α-galactosidase activity) as substrate. (Ia) Residual α-GalNAcase activity of enzyme I; (Ib) residual α-galactosidase activity of enzyme I; (II) residual α-GalNAcase activity of enzyme II. Data are presented as mean ± MD (*n* = 3)

## Discussion

In a previous study we successfully distinguished α-GalNAcase I and II from starfish with respect to their enzymatic properties [19]. Like typical form of α-GalNAcases isolated from various animal tissues, α-GalNAcase I contained intrinsic α-galactosidase activity [7, 9–12]. Contrary, α-GalNAcase II was free from α-galactosidase activity and rare in animal tissues. α-GalNAcase without α-galactosidase activity was available in microbes [13–17]. The subunit mass of α-GalNAcase I and II appeared to differ on SDS-PAGE in native form and after deglycosylation with PNGase F (Fig. 1a). The differences in subunit mass and enzyme kinetics of α-GalNAcase II with α-GalNAcase I highlighted the possibility that this is an exceptional form in animal sources.

Immunological study with antibody raised against human α-GalNAcase clearly distinguished the two forms. Isoform I intensely reacted with the antibody, whereas isoform II had no detectable reactivity (Fig. 1 b-1). As expected, the same results were obtained when tested with anti-squid α-GalNAcase I (Fig. 1 b-2). In contrast, anti-squid α-GalNAcase II reacted equally with α-GalNAcase I and II (Fig. 1 b-3). These results suggest the presence of both common and different antigenic determinants on both α-GalNAcase I and II and indicate their similarities and differences in structure. The structural interrelationship between α-GalNAcase I and II was clearly understood from their N-terminal sequencing and peptide mapping (Table 1 and Fig. 2). The high level of sequence similarities of the N-terminal amino acids and the internal peptides of α-GalNAcase I and II with chicken and human α-GalNAcase allowed us to locate the structural conservation site including the active site and the substrate recognition loop (Table 1 and Figs. 3, 4). Collectively, the results suggest that α-GalNAcase I and II are closely related with regard to their primary and higher order structure and belong to GHF 27 glycosidase [8].

**Fig. 6:**
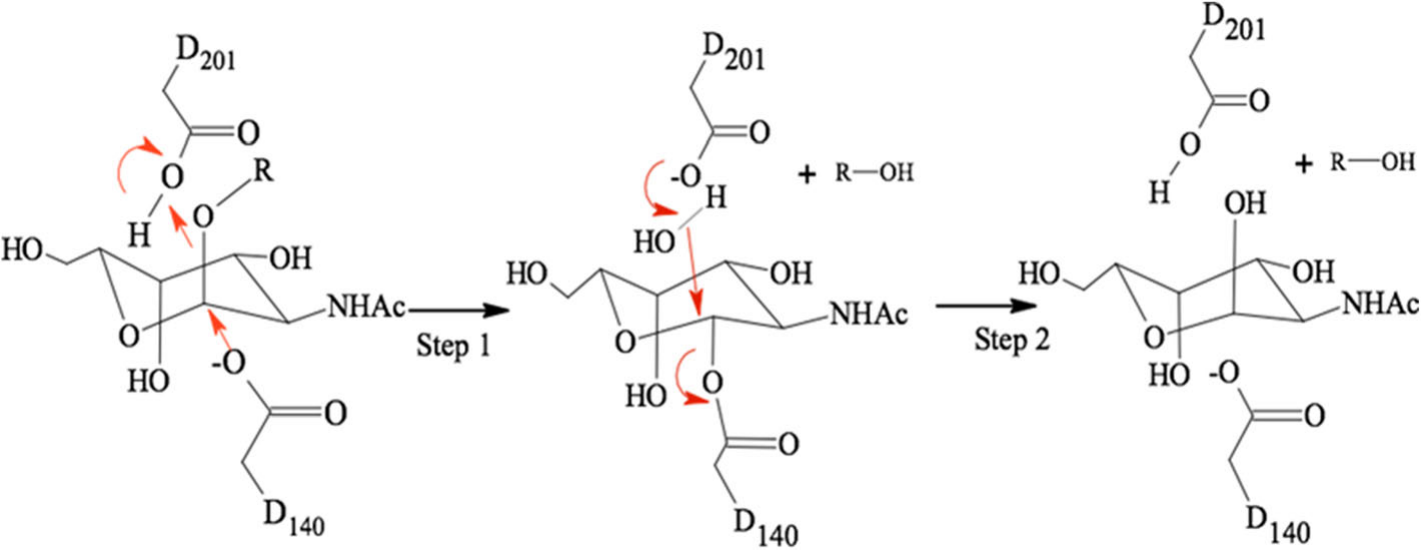
Double displacement catalytic mechanisms of starfish α-GalNAcase I and II. Step 1: D140 from α-GalNAcase makes a nucleophilic attack on C1 of terminal α-GalNAc of substrate, cleaving the glycosidic linkage and producing a covalent intermediate complex. Step 2: A water molecule, deprotonated by D201, makes a nucleophilic attack on C1 of α-GalNAc and cleave the covalent enzyme-substrate intermediate complex and release N-acetylgalactosamine

**Fig. 7:**
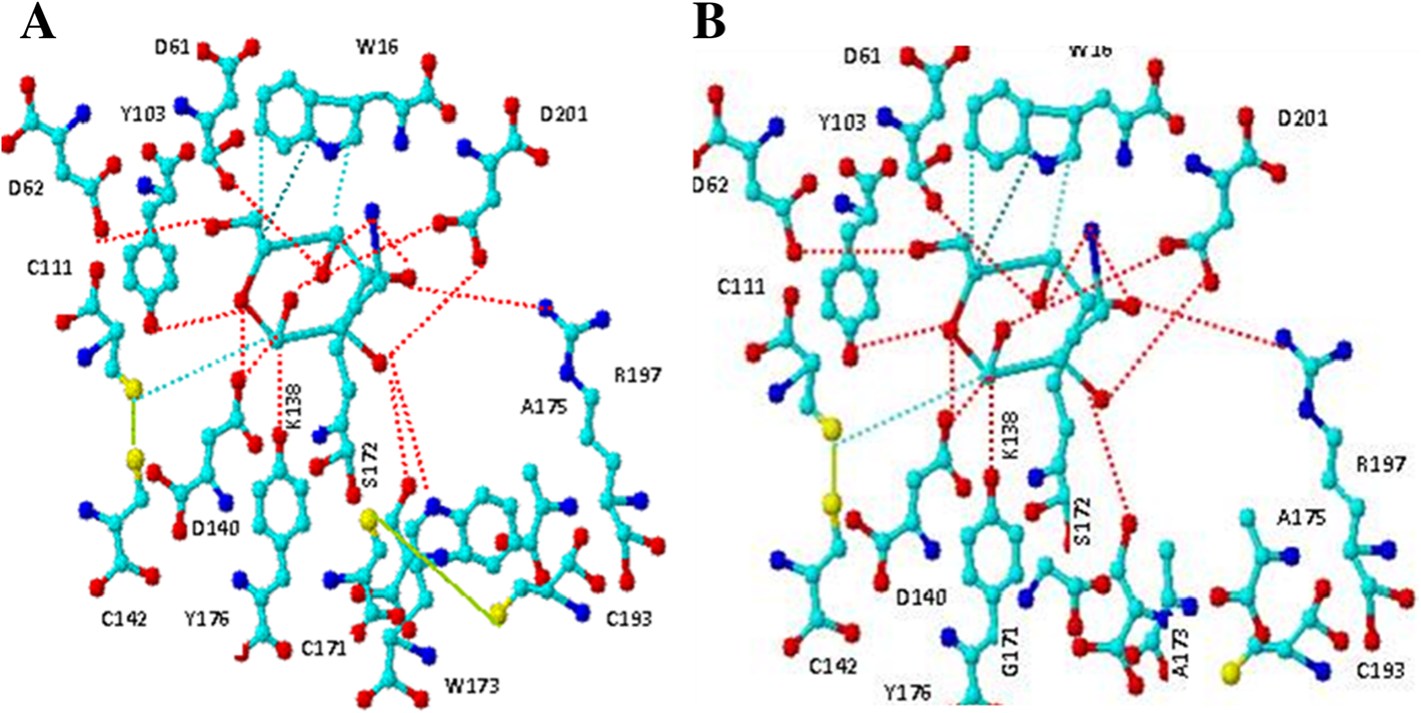
Proposed interactions between ligand and active site residues of α-GalNAcase I and II. The α-GalNAc ligand was placed on the center of active site and the interactions between ligand and active site residues were analyzed by software ACDLabs Free software 5.0. The ligand is surrounded by amino acid side chains labeled in the active site of α-GalNAcase I **a** and α-GalNAcase II **b**. The hydrogen bond and polar interactions are shown in red and hydrophobic interactions are shown in blue

α-GalNAcase is a retaining glycosidase, where both substrate and product have anomeric carbons with α configurations. The catalytic mechanism of α-retaining GHF 27 members is based on a double displacement catalytic reaction by two conserved aspartate residues [26, 28]. The mechanism involves first the nucleophilic attack of D140, and D201 serves as a subsequent proton donor and acceptor by the carboxyl group on the α1 position of ligand (Fig. 6). The active site residues W16, D61, D62, Y103, C111, K138, D140, C142, S172, A175, Y176, R197 and D201 are conserved in α-GalNAcase homologues series [12, 15, 21, 22, 29, 30]. Among the 13 residues, 11 are common in human α-galactosidase, and the other two define the substrate specificities between α-GalNAcase and α-galactosidase [31]. The residues S172 and A175 of the substrate recognizing loop (SCS_172_WPAY_176_), also called *N*-acetyl recognition loop, in α-GalNAcase are replaced with glutamate and leucine, respectively in human α-galactosidase A, to accommodate smaller hydroxyl group of α-galactosyl ligand but not the larger N-acetyl group [26, 31]. In α-GalNAcase II, we have identified substitution of two conserved residues, G171 and A173 in place of the corresponding residues, C171 and W173 in typical α-GalNAcases (Fig. 4). Since the residues are located in the substrate recognizing loop, it is highly possible that they might influence the recognition of the substrate. From molecular modeling, It was observed that the disulfide bridge (C171-C193), which is formed in typical α-GalNAcases and involved in hydrophobic interaction with C-2 of α-galactosyl ligand, is disrupted in α-GalNAcase II due to replacement with G171 (Fig. 4, Fig. 7a). The large and bulky W173 is replaced with a small residue alanine within the loop (SGS_172_APAY_176_) in α-GalNAcase II and thus generate a larger space for the pocket for accommodation of the N-acetyl group as compared to typical α-GalNAcases. Similarly, the small hydroxyl group of galactose moiety loosely fits in the loop cavity and hence showed no or too little α-galactosidase activity in absence of a bulkier indole ring of tryptophan (Fig. 7b). This is supported by N-acetyl recognition loop composition (−SDSAPAY-) of *Acremonium species* α-GalNAcase which hardly showed α-galactosidase activity [15].

Chemical modification studies supported the presence of serine, aspartate and tryptophan residues in the active site of α-GalNAcase (Table 2). A single tryptophan residue (W16) was found to be involved in α-GalNAcase activity, while two tryptophan residues (W16 and W173) was involved in α-galactosidase activity of α-GalNAcase I (Fig. 5a). Inhibition of α-GalNAcase and α-galactosidase with GalNAc supports the existence of dual activities of α-GalNAcase I (Fig. 5b), because GalNAc is the specific inhibitor of α-GalNAcase enzyme [12, 32]. The second tryptophan (W173) is replaced with alanine in α-GalNAcase II and showed to be free from α-galactosidase activity.

## Conclusion

In conclusion, the experimental results obtained and discussed in this study clearly suggest that α-GalNAcase I and II are closely related with respect to their primary and higher order structure. Structural comparisons of GalNAcase I with GalNAcase II provided understanding of their differences in substrate specificities in these enzymes. Further studies are necessary to find out and isolate genes, which encode two catalytically different α-GalNAcases evolved from a common ancestor.

## Methods

### Materials

*p*-Nitrophenyl-α-N-acetylgalactosaminide (pNP-α-GalNAc), pNP-α-galactoside and PNGase F were purchased from Sigma Aldrich Chemical Co (Stelnhelm, Germany). Di-isopropyl-fluorophosphate (DFP), carbodiimide cyanamide HCl (CNM-HCl), N-bromosuccinimide (NBS), bovine serum albumin (BSA) and lysylendopeptidase were procured from Wako Pure Chemical Industries (Osaka, Japan). Peroxidase conjugated sheep anti-rabbit IgG was obtained from Organon Teknika Corporation (Philadelphia, USA). Unless otherwise directed, all other chemicals and reagents were of analytical grade and obtained from commercial sources.

### Purification and characterization of α-GalNAcase from starfish

The purification and enzymatic characterization of α-GalNAcase I and II from digestive organ of starfish were done by the procedures as reported [19]. Enzyme activity was determined by the technique as described previously [33]. Protein concentration was determined by bicinchoninic acid (Sigma) assay with BSA as standard [34]. Sodium duodecyl sulfate polyacrylamide gel electrophoresis (SDS-PAGE) was performed on 12.5% gel according to the procedure described by Laemmli [35]. Proteins were visualized on SDS-PAGE with Coomassie brilliant blue (CBB) R-250 [36].

### Deglycosylation of α-GalNAcase I and II

The purified α-GalNAcase I and II (10 μg) were denatured in 50 μl sodium phosphate (50 mM) buffer, pH 7.0 by heating at 100 °C for 10 min in presence of 0.02% SDS and 10 mM β-mercaptoethanol. The denatured protein was treated with Triton X-100 (Conc. 1.5%) and incubated at 37 °C with 1 unit of PNGase F (Sigma) for 24 h. The PNGase F treated enzyme were separated on SDS-PAGE and stained with CBB R-250 solution.

### Electro-blotting and immune-staining

Purified enzyme (5 μg) was separated on SDS-PAGE and transferred to polyvinyledine fluoride (PVDF) membrane (Millipore, USA) by electro-blotting in a Biorad mini-trans blot cell using Tris-glycine buffer. Nonspecific binding sites of membrane were saturated by incubation with 3% BSA in 50 mM Tris–HCl containing 150 mM sodium chloride and 0.1% sodium azide (buffer A) for 1 h at room temperature. The membrane was then incubated with primary antibody at an appropriate dilution for 30 min. After rinsing, the membrane was incubated for 1 h with peroxidase conjugated sheep anti-rabbit IgG in buffer A. The protein which crossreacted with the antibody was visualized using 3, 3-diaminobenzidine (DAB) intensified with 0.001% hydrogen peroxide.

### Protein digestion and peptide mapping

Purified enzyme (100 μg) was separated on SDS-PAGE and visualized by CBB staining. The expected protein band was excised with a sharp blade and washed with water. The gel containing protein band was cut into pieces and destained with 40% acetonitrile in 100 mM ammonium carbonate for 24 h. Then protein in gel was reduced with DTT (10 mM) at 56 °C and alkylated with iodoacetamide (50 mM) in 100 mM ammonium carbonate for 1 h in every treatment. The polyacrylamide gel was dehydrated with acetonitrile after completion of each treatment. The reduced and alkylated proteins were digested with lysylendopeptidase at protein to peptidase ratio 50:1 in 50 mM Tris–HCl buffer, pH 8.5 for 24 h. After digestion, the peptides were extracted from polyacrylamide gel once with 1.0% formic acid, once with 9% acetonitrile in 20 mM ammonium carbonate and twice with 60% acetonitrile in 0.1% formic acid. The extracted peptides were lyophilized by freeze drying, reconstituted in water and subjected to RP-HPLC separation on a column TSK gel ODS-120 T (4.6 × 150 mm, Tosoh, Tokyo, Japan) at a flow rate 1 ml min^−1^ followed by elution with a 90 min linear gradient of acetonitrile (5-50%) in 0.1% trifluroacetic acid (TFA). The peptide peaks were detected at 215 nm.

### MS and MS/MS analysis of peptides

The individual peptide fractions obtained from RP-HPLC separation were adsorbed on PVDF membrane by repeated pipetting of the sample solution. The resulting bound samples were desalted with 10 μl 0.1% TFA and spotted on a MALDI target plate that was double layered with 2, 5-dihydroxybenzoic acid (10 mg/ml in acetone). The spots were air dried and MS/MS fragmentation was performed using a MALDI-TOF-MS Autoflex (Bruker Daltonics, Billerica, MA, USA) under a 20 kV accelerating voltage in the positive ion mode. The Bruker Data Analysis software (Version 4.0) was employed for data acquisition, processing and analyses. The mass tolerance in all experiments was ≤ ±0.4 Da.

### Amino acid sequencing

Purified enzyme α-GalNAcase I and II (10 μg each) was resolved on SDS-PAGE and transferred to PVDF membrane by electro-blotting. The protein was stained with 0.1% Ponceau S (Sigma) in 5% acetic acid. After staining, expected protein band was excised and washed with methanol and subjected to sequence the N-terminal amino acids [15, 16], with a automated gas-phase protein sequencer (Shimadzu, Kyoto, Japan), followed by phenylthiohydantoin analysis with HPLC. Internal amino acid sequence derived from lysylendopeptidase digested peptides of α-GalNAcase I and II deduced by MS/MS analyses were confirmed by the amino acid sequence analyzer.

### Chemical modification studies

Chemical modifications were performed by incubating the enzyme with DFP in 20 mM Tris–HCl, pH 4.0, and CNM-HCl and NBS in 50 mM citrate-phosphate buffer, pH 4.0 (CPB). Purified enzyme (45 μg per ml) was incubated at 25 °C in various concentrations of modifying agent for 5 min [25], followed by estimation of residual activity. The residual activity was determined against pNP-α-GalNAc (for α-GalNAcase activity) and pNP-α-galactoside (for α-galactosidase activity) as substrate. To determine the number of tryptophan residues involved in enzyme activity, purified α-GalNAcase I (5 μM) was incubated with NBS (0–60 μM) at 25 °C for 5 min in CPB. NBS modified inactivation was monitored spectrophotometrically by measuring the decrease in absorbance at 280 nm. The numbers of tryptophan residues modified were determined using extinction co-efficient of 5500 M^−1^cm^−1^ [27]. Modification reaction was blocked by adding excess L-tryptophan (50 mM) followed by determination of residual activity.

### Molecular modeling for the interaction between ligand and active site residues of α-GalNAcases

The deduced peptide sequences of starfish α-GalNAcase I and II were substituted in chicken α-GalNAcase (PDB code: 1Ktb) aligned sequence and constructed a fold fit structure by DeepView/Swiss-PdbViewer based on the chicken α-GalNAcase structure as a template [26]. From the modelling we designed the catalytic site of α-GalNAcase I and α-GalNAcase II. The α-GalNAc ligand was placed on the centre of active site and the interactions between ligand and active site residues were analyzed by ACDLabs Free software 5.0.

## Abbreviations

DAB: 3,3-diaminobenzidine
DTT: 1,4-dithiothretol
Gal: D-galactose
GalNAc: D-N-acetylgalactosamine
GHF: glycoside hydrolase family
Glc: D-glucose
GlcNAc: D-N-acetylglucosamine
Man: mannose
α-GalNAcase: α-N-acetylgalactosaminidase
